# Behavioral determinants for vaccine acceptability among rurally located college students

**DOI:** 10.1080/21642850.2018.1505519

**Published:** 2018-09-06

**Authors:** Rebecca K. Britt, Andrew M. Englebert

**Affiliations:** a Department of Journalism and Creative Media, College of Communication & Information Sciences, The University of Alabama, Tuscaloosa, AL, USA; b AME Family Law Firm, LLC, Appleton, WI, USA

**Keywords:** Rural populations, vaccination acceptability, theory of planned behavior, vaccination uptake

## Abstract

**Background:** College-aged adults in a rural and medically-underserved area often struggle to receive proper vaccinations due to lower socioeconomic status coupled with life demands.

**Objectives:** The Theory of Planned Behavior (TPB) was used as the theoretical basis to explore behavioral determinants associated with vaccination uptake in the population.

**Methods:** This study used a questionnaire distributed to college students (*n* = 208) located in a rural area to assess the effects of social and behavioral factors on vaccination uptake.

**Results:** Attitudes and normative beliefs towards vaccination uptake were positive but were largely impacted by work demands. Perceived behavioral control did not contribute towards the intent to receive necessary vaccines.

**Conclusions:** Researchers conducting vaccination interventions, along with physician-patient communication, need to target attitudes and subjective norms in rural and medically underserved communities to increase vaccines, particularly HPV. In addition, results showed that promoting vaccine uptake among minorities is necessary to aid in vaccine acceptability in these communities.

## Introduction

Vaccination programs are among the most successful health initiatives across the United States, with a longstanding body of scientific evidence and public support. Vaccinations are considered to be widespread public health achievements that save lives and improve quality of life by preventing infectious diseases (National Vaccine Program Office, 2015). Despite this, the success of obtaining vaccinations has been challenged for a variety of reasons; among them including contextual factors (Jain, van Hoek, Boccia, & Thomas, 2017; Larson, Jarrett, Eckersberger, Smith, & Paterson, 2014; Lu et al., 2017). For instance, vaccine hesitancy has been identified in rural and medically underserved communities, often due to barriers including lower socioeconomic status and income (e.g. Bennett, Pumkam, & Probst, 2011; Wu et al., 2008) and recently, gender identity (Bednarczyk, Whitehead, & Stephenson, 2017). Medically-underserved citizens located in rural areas tend to face challenges for vaccination administration due to fewer available healthcare providers, increased rates of poverty, and higher rates of uninsurance (National Rural Health Foundation, 2015). In particular, college-age adults in a rural and medically-underserved area may struggle to get proper vaccinations, particularly when they have competing demands, such as family, social, school, and work obligations that consume their attention and their time. Partnerships among states and universities have emerged to develop strong medical students, but studies of these populations-at-large remain necessary to improve vaccination uptake and help build healthier communities (Florence, Goodrow, Wachs, Grover, & Olive, 2007).

This study investigated the demands of family, school, social, and work and the potential relationships and their potential impact with attitudes, subjective norms, and perceived behavioral control (Ajzen, 1991) related to vaccination uptake. These findings can aid in determining how to develop and promote vaccination-based interventions for rurally located young adults, to increase education about vaccinations based on various aspects of attitudes, norms, and perceived control, and to aid in building public health interventions that can address these needs based on this information.

## Literature review

In 2016, there were more than 40 million people in the U.S. with incomes below twice the federal poverty level (U.S. Census Bureau, 2017) who also lacked insurance, a widely-recognized problem. Rural and medically underserved areas typically refer to people who lack adequate and primary health care coverage (Rosenbaum, Jones, Shin, & Ku, 2009). Medical underservice designations are used to help distribute federal and state funds to residents with especially high health care needs, which includes colleges in poorer areas. However, even with these numbers, medically underserved areas also have age, poverty, and health status that are difficult to fully take into account (Ricketts et al., 2007). This includes vaccination uptake, which tends to be lower in these areas, but previous research has shown successful programs that have helped to incentivize healthcare promotion (Hirpa et al., 2016). Current research shows that low vaccine uptake tends to be predicted by living in deprived areas or alone (Jain et al., 2017).

Previous research on rural communities have found that understanding citizens’ behaviors are critical to building healthier neighborhoods (e.g. Benitez, Keller, Coe, & Tasevska, 2017; Chumbler, Hartmann, Cody, & Beck, 2001; Elnitsky & Alexy, 1998) and to reduce emergency healthcare needs (Simmons, Anderson, & Braun, 2008). Studies that have examined the specific vaccines that college-aged young adults receive have noted that they tend to have basic knowledge, that they tend be in the early decision-making phases relating to vaccinations (Barnard, George, Perryman, & Wolff, 2017), and that rurally located adolescents and young adults who receive vaccines and routine healthcare visits are more likely to help increase vaccine coverage in their communities (Reiter, McLee, Gottlieb, & Brewer, 2011).

Perceptions of and vaccination uptake practices among college-aged adults have been explored in a variety of contexts. In some studies, college students have been less likely to be vaccinated unless a mandate was instituted (Looper, George, Johnson, & Conway, 2017). According to the American College Health Association (2017), recommended vaccines among college students include influenza, tetanus, HPV, Hepatitis A, Hepatitis B, Measles, Polio vaccine, Varicella, Meningococal vaccine, and Rubella. The ACHA notes that some vaccines are recommended annually (e.g. influenza), while others might be administered based on populations at risk (e.g. meningococcal is recommended for students living in residence halls or if they are working in laboratories). Broadly speaking, among the most commonly needed vaccines for college students recommended by the ACHA (2017) and reported as received include influenza (Adams, Canclini, & Tillman, 2018; Huang et al., 2018; Shropshire, Brent-Hotchkiss, & Andrews, 2013), HPV (Catalano et al., 2017; Paiva, Lipschitz, Fernandez, Redding, & Prochaska, 2013), meningococcal (Banzhoff, 2017), and varicella (Jewett et al., 2016). Although HPV is widely regarded as a necessary vaccine, many barriers have been noted towards completion, such as perceived cost and usefulness (Paiva et al., 2013), that female gender tends to be a predictor of completion (Bednarczyk et al., 2017; Richman, Maddy, Torres, & Goldberg, 2016), access to care remains a paramount issue (Barnard et al., 2017), and several others. Barriers to obtaining vaccines likewise often include cost (Tran et al., 2017), patients failing to make routine healthcare visits, perceived psychosocial barriers, and in the case of rural college-aged adults, increased life demands (Scott, Miller, & Morris, 2016; Webber & Boehmer, 2008).

For young adults in a medically underserved area, the demands of family, social, school, and work obligations (Scott et al., 2016) often act as additional barriers toward immunizations, even as calls for investments to improve public health in rural areas have increased (Rosenbaum et al., 2009). For young adults in a rural, medically underserved area, particularly those in college, several challenges exist, which further require researchers to examine daily life demands alongside their vaccination behaviors. Researchers have argued that a problem facing rural students entering college is a poor college preparation, and many students coming from first generation backgrounds, requiring additional financial aid, additional time spent working, and facing other challenges, which can inhibit the need to seek out healthcare (Webber & Boehmer, 2008). Students in a rural area may be less likely to get any necessary vaccinations if they are facing increased stresses and time consumption from family, social factors (Wheelock, Thomson, & Sevdalis, 2013), school, and work obligations.

Vaccine needs in rural areas have been addressed through grassroots partnerships via state sponsored programs to bring together resources to develop rural health care networks, such as in the state of Georgia (Minyard, Lineberry, Anderson-Smith, & Byrd-Roubides, 2003), and have likewise examined factors such as the impact of gender identity to understand HPV vaccine uptake (Bednarczyk et al., 2017). School based programs have been found to be successful for initiating necessary vaccinations among young adults such as those in high school and college (Vanderpool et al., 2015). In a rural area in Kentucky, a program was implemented in a school system to promote HPV vaccination through programmatic measures, including parental consent and offering the vaccine (Vanderpool et al., 2015). Briefly put, results of this program offered evidence of success for school-based immunization. Similarly, studies have been conducted in colleges to assess the eligibility and willingness of students to participate in ‘catch up’ programs to promote the vaccine (Richman, Haithcox-Dennis, & Allsbrook, 2012), along with programs that promote vaccines among general school systems (Tran et al., 2017), finding that these are successful.

### Theory of planned behavior

Behavioral theories can facilitate how a population might accept or deny healthy behaviors (e.g. Wiemken et al., 2015). To address the attitudes towards vaccines, perceptual factors, and perceived ability, along with intent to get vaccinated, the Theory of Planned Behavior (TPBC; Ajzen, 1991; Fishbein & Ajzen, 1975) offers a well-rounded framework often used in health psychology research. TPB outlines that behavioral decisions are guided by three determinants: attitudes, subjective norms, and perceived behavioral control. These determinants capture and help to predict influences on behavior in the decision-making process (Ajzen, 1991). Attitudes refer to the favorable or unfavorable evaluations one places on the consequences of a behavior. In planned behavior research, attitudes are consistently a strong predictor of behaviors (Hagger, Chatzisarantis, & Biddle, 2002; McEachan, Conner, Taylor, & Lawton, 2011). Subjective norms refer to the beliefs about the expectations of significant others (such as family or friends) regarding whether or not performing a behavior is desirable. Perceived behavioral control (PBC) refers to the perception of the ease or difficulties regarding the behavior at hand, or any limitations that may inhibit the behavior. It may be expected that an individual who has already completed a course of vaccinations would have very high perceived behavioral control, as he or she has already demonstrated the ability to receive those vaccines. In the current study, perceived behavioral control is conceptualized in the context of confidence and certainty of being able to receive appropriate vaccines (Yzer, 2012).

### Hypotheses

As outlined through TPB constructs, we posit that rural college students’ attitudes, subjective norms, and perceived behavioral control regarding vaccination are related to one another as well as the intent to receive vaccinations. It is also expected that behavioral intent may be linked with actual vaccination uptake. Additionally, the anticipation that external pressures—particularly the family, social, school, and work demands that rural students face—will impact their attitudes, subjective norms, and perceived behavioral control, ultimately impacting vaccination behaviors through those TPB variables. With this in mind, the following three hypotheses are proposed:

H1: Rural students’ family, social, school, and work demands are associated with their attitudes, subjective norms, and perceived behavioral control related to vaccination intent.

H2: Rural students’ attitudes, subjective norms, and perceived behavioral control are associated with their intent to receive vaccines.

RQ1: What vaccines do rural students’ report as being received?

## Methods

### Study design

Upon Institutional Board Review approval, the current study was conducted via a survey administered in Qualtrics® at a rural university in the United States. As of January, 2017, the area surrounding the university was federally designated as medically underserved (HRSA Data Warehouse, 2017), including an area with a shortage of primary healthcare providers, and some specialty providers. The closest healthcare centers that provide specialty needs beyond primary care (e.g. orthopedics, rheumatology, and so on) are located an hour away via driving distance.

The survey was designed to ask participants about their attitudes, subjective norms, perceived behavioral control, and behavioral intent related to vaccination based upon Ajzen’s (2015) recommendations on constructing a TPB questionnaire. Subjects were also asked what vaccinations they had received since turning 18, using a list of appropriate vaccines compiled from recommendations from the Centers for Disease Control and Prevention for individuals older than 18. Finally, participants were asked about the frequency with which family, social, school, and work obligations placed demands on their time.

### Participants

All participants were required to be over the age of 18 to participate in the study. Participants who indicated that they were under 18 years old were redirected to another link offering them other survey opportunities for which they would be eligible. A college-wide research participation system allowed participants to select a study to participate in as part of a requirement to engage in research activities in exchange for college credit, with this being one of the options available. Participants had the option to opt out at any time.

While several vaccines are not administered beyond the age of 26, questions in the survey utilized Ajzen’s original items (e.g. attitude, normative beliefs, intent) to gauge the relationship between TPB variables and vaccination behaviors. Vaccinations outlined in the current article are those recommended by the Centers for Disease Control and Prevention after the age of 18 (2017). A total of 211 individuals responded to the survey. Of these responses, 3 were incomplete or contained missing values. These were removed, leaving a total of 208 observations that were included in this study. Participants included slightly more females (59.0%) than males (41.0%). The vast majority of students reported their ethnicity as Caucasian (86.2%), followed by Native American (8.0%), Asian (2.3%), African American (1.1%), Hispanic/Latino (1.1%), Indian (1.0%), or undisclosed (0.3%). Participants identified their year in college as freshmen (42.5%), sophomores (41.5%), juniors (0.4%), or seniors (6.6%). The majority of participants reported their home as being in a rural area (76.0%), with the remaining reporting living in a suburban area (24.0%).

### Measures and variables

#### Attitudes towards vaccinations

In the current study, it would be expected that individuals with a positive attitude towards vaccination would be more likely to engage in getting necessary vaccines. Three items comprised attitudes towards vaccinations themselves, measured through the averages using a 7-point Likert scale, where 1 = *strongly agree* and 7 = *strongly disagree*. Items included, ‘I believe it is beneficial to obtain any necessary vaccinations,’ ‘I think I should get any vaccinations I might need,’ and ‘In general, how do you feel about getting any vaccinations needed?’ (*M *= 4.21, *SD *= 1.46). These items showed high internal consistency (Cronbach’s alpha = .93).

#### Subjective norms towards vaccinations

Subjective norms were measured through the average of four items, e.g. ‘Most people who are important to me (family, friends, coworkers) think I should get any necessary vaccines this year.’ All four items were presented as 7-point Likert scales, where 1 = *strongly agree* and 7 = *strongly disagree* (*M *= 5.50, *SD *= 1.41). The subscale showed good internal consistency (Cronbach’s alpha = .92).

#### Perceived behavioral control towards vaccinations

PBC was measured through the average of three 7-point Likert scale items (e.g. ‘Whether or not I get vaccinations is completely up to me’) for which 1 = *strongly agree* and 7 = *strongly disagree* (*M *= 4.94, *SD *= 2.07). These items showed good internal consistency (Cronbach’s alpha = .90).

#### Behavioral intention towards vaccinations

Behavioral intention was measured through the average of three items (‘I intend to get any vaccinations I may need this year,’ ‘I plan to get any vaccinations I may need this year,’ and ‘I want to get any vaccines I may need this year’). These questions were likewise administered on a 7-point Likert scale where 1 = *strongly agree* and 7 = *strongly disagree* (*M *= 4.28, *SD *= 1.46). This subscale also showed good internal consistency (Cronbach’s alpha = .91).

#### Family, social, school, and work demands (control beliefs)

Four questions were constructed based on Ajzen’s TPB questionnaire (2015) as direct measures of control beliefs. These questions used 7-point bipolar adjective statements ranging from ‘very rarely,’ to ‘very frequently.’ The four questions were as follows: ‘How often do family obligations place unanticipated demands on your time?’ (*M *= 3.70, *SD *= 1.37), ‘How often do your friends, significant other or co-workers place unanticipated demands on your time?’ (*M *= 4.15, *SD *= 1.31), ‘How often do your classes place unanticipated demands on your time?’ (*M *= 5.51, *SD *= 1.33), and ‘How often does work or employment place unanticipated demands on your time?’ (*M *= 3.83, *SD *= 1.77).

#### Measuring vaccination behavior

The American College Health Association (2017) and the CDC (2017) report several vaccines (Table 4) recommended for young adults. Consistent with the ACHA’s phrasing (2017), participants were asked 8 questions on their vaccination behavior, e.g. ‘Have you received the following vaccinations (shots or series of shots)?’ with 3 response choices, as outlined by the ACHA, including ‘Yes,’ ‘No,’ and ‘Don’t know.’

### Statistical analysis

SAS was used to conduct all statistical analyses. Hypotheses were primarily tested via a confirmatory factor analysis using the hypothesized relationships among the given factors and TPB variables. A confirmatory factor analysis (CFA) was used to assess the importance of each item on the TPB constructs. The model fit was assessed using a chi-square (*X*^2^) test, Comparative Fit Index (CFI), Root Mean Square Error of Approximation (RMSEA), Incremental Fit Index (IFI), and Bentler-Bonett Normed Fit Index (NFI). Recommendations from Kenny (2014) suggest that CFI, IFI, and NFI values of >.90 are acceptable and an RMSEA of .05 to be a good fit. The model fit index revealed the following scores: *X*^2^ (206) = 140.327, *p* = .000; RMSEA = .052, CFI = .921, IFI = .913, NFI = .901.

Pearson correlations were calculated to assess the relationship between the TPB items and constructs. The alpha level for testing significant correlations was set to *α* = .01. This was done in order to mitigate the inflated risk of a Type 1 error due to the number of correlations being examined. Attitudes, subjective norms, perceived behavioral control, and behavioral intent were assessed as omnibus scores using the sum of all items within their respective subscales. Each of the eight vaccination behaviors was assessed independently in order to observe any possible differences in the adoption of different vaccinations. Variables were *z*-transformed prior to analysis, and all hypotheses were tested at the *α* = .05 significance level.

## Results

**Figure 1.**
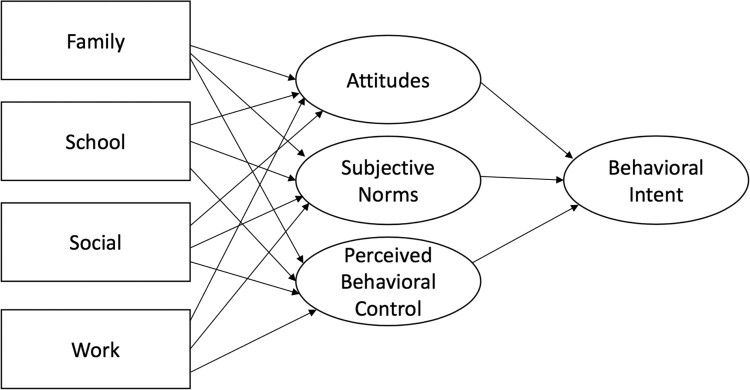
Proposed model of family, school, social and work demands, TPB variables and behavioral intent to get vaccinated.

H1 stated that rural students’ family, social, school, and work demands are associated with their attitudes, subjective norms, and PBC related to vaccination. To address H1, CFA (Table 1), and Pearson correlation coefficients were conducted addressing family, social, school, and work variables and TPB constructs (Table 2). The fully proposed model based on the results of the study is shown in Figure 1. The model uses the manifest variables of family, school, social, and work demands along with latent TPB variables. Prior health research using cross-sectional data have built models that include latent and manifest variables (e.g. Lu et al., 2014). Results showed that attitudes towards vaccination uptake were positively related to increased work demands (*r* = .223, *p* < .001). Subjective norms did not have a significant relationship at the *p* < .01 level associated with any variable in the study. PBC and vaccination uptake was associated with work demands (*r* = .168, *p* < .001), school demands (*r *= .227, *p* < .01), and social demands (*r* = .056, *p* < .001). Intent was predicted by work demands (*r* = .143, *p* < .01), school demands (*r* = .130, *p* < .01), and social demands (*r* = .080, *p* < .01). The relationship among these variables suggests that vaccination intent is largely predicted by demands on rural students’ time. For work, school, and social demands, there was a negative relationship with receiving vaccinations.

**Table 1.** Confirmatory factor analysis of TPB variables and family, social, school, and work variables.

Path	Effect	Std. Error	*t* value	*p* value	
Family -> Attitudes	0.0244	0.1120	0.2178	0.8276	
Family -> Subjective norms	0.0346	0.1143	0.3027	0.7622	
Family -> PBC	−0.0885	0.1117	−0.7920	0.4284	
Social -> Attitudes	−0.0556	0.1218	−0.4562	0.6482	
Social -> Subjective norms	0.0520	0.1244	0.4183	0.6757	
Social -> PBC	−0.0228	0.1219	−0.1871	0.8516	
School -> Attitudes	0.2461	0.0967	2.5454	0.0109	*
School -> Subjective norms	0.0799	0.1014	0.7880	0.4307	
School -> PBC	0.2353	0.0970	2.4270	0.0152	*
Work -> Attitudes	−0.0828	0.1114	−0.7428	0.4576	
Work -> Subjective norms	−0.1597	0.1129	−1.4143	0.1573	
Work -> PBC	−0.0614	0.1116	−0.5500	0.5823	
Attitudes -> Intent	0.3890	0.0966	4.0263	<0.0001	*
Subjective Norms -> Intent	0.2017	0.0874	2.3066	0.0211	*
PBC -> Intent	0.2953	0.0918	3.2236	0.0013	*
Intent -> Tetanus	0.2494	0.0947	2.6324	0.0085	*
Intent -> Measles	0.4241	0.0828	5.1188	<0.0001	*
Intent -> HPV	0.3584	0.0880	4.0707	<0.0001	*
Intent -> Hepatitis A	0.4764	0.0781	6.1011	<0.0001	*
Intent -> Hepatitis B	0.4531	0.0803	5.6447	<0.0001	*
Intent -> Polio vaccine	0.4363	0.0818	5.3347	<0.0001	*
Intent -> Meningococcal	0.4658	0.0791	5.8894	<0.0001	*
Intent -> Varicella	0.4046	0.0845	4.7894	<0.0001	*

*Significant relationships at the *α* = .05 level.

**Table 2.** Pearson correlation coefficients among work, school, and social demands and planned behavior variables on vaccination intent.

	Work demands	School demands	Social demands	Attitudes	Subjective norms	Intent	Perceived behavioral control
Work demands	1						
School demands	.076	1					
Social demands	.490**	.194*	1				
Attitudes	.223*	.014	.007	1			
Subjective norms	.187	.160	.044	.451	1		
Intent	.143*	.130*	.080*	.425	.766	1	
Perceived behavioral control	.168**	.227*	.056*	.424	.739	.793	1

Note: *p <* *.01; **p < . 001; Work Demands (*M *= 3.81; SD = 1.76); School Demands (*M *= 5.50; SD = 1.32); Social Demands (*M *= 4.14; SD = 1.30).

H2 stated that rural students’ attitudes, subjective norms, and PBC are associated with their intent to receive vaccines. In the regression model, vaccination intent was used as the dependent variable, with attitudes, subjective norms, and perceived behavioral control included as independent variables in the model. The overall regression was significant, *F*(3, 202) = 77.68, *p* < .05, *R*^2^ = .686*.* Attitudes (*p *< .001) and subjective norms (*p *< .01) were statistically significant predictors of vaccination intent. Perceived behavioral control, however, did not significantly contribute to the model (*p *= 0.36), and was not related to vaccination intent (Table 3).

**Table 3.** Summary of regression model of vaccination intent based on TPB variables.

	B	SE_B_	*β*	*t*	*p*
Attitudes	0.45	0.09	0.59	1.45	.001**
Subjective norms	0.33	0.07	0.12	1.71	0.01*
Perceived behavioral control	0.57	0.08	0.16	1.92	0.36

Note: **p < .001; *p < .01.

RQ1 asked what vaccines were reported by rural students as being received. Of those recommended by the CDC, there was a disparity reported by participants. Among the most prevalent included tetanus (86.0% of men and 76.2% of women reported receipt), MMR (65.1% of men; 66.7% of women) and Hepatitis B (53.5% of men; 58.7% of women). Overwhelmingly, the lowest vaccine obtained was HPV (14.2% of men; 30.2% of women). These results, however, are consistent with reports from the American College Health Association (2017). Table 4 shows the full results. Implications of these results are reviewed in the discussion.

**Table 4.** Vaccines reported as received among rural college students.

	Men			Women		
	Received (%)	Mean	(*SD*)	Received (%)	Mean	(*SD*)
Tetanus	86.0%	1.14	0.35	76.2%	1.20	0.40
Rubella	65.1%	1.35	0.48	66.7%	1.30	0.46
HPV	14.2%	1.50	0.50	30.2%	1.42	0.49
Hepatitis A	48.8%	1.47	0.50	55.6%	1.38	0.49
Hepatitis B	53.5%	1.58	0.50	58.7%	1.38	0.49
Polio vaccine	41.9%	1.58	0.49	47.9%	1.50	0.50
HIB-MenCY	42.9%	1.58	0.46	46.0%	1.52	0.50
Varicella	30.2%	1.70	0.46	38.1%	1.60	0.49

Note: *N* = 208.

## Discussion

This study sought to explore the vaccination behaviors of rurally located college students in a medically underserved community. This was done in light of previous research on vaccine practices that has demonstrated the need to explore rural communities’ efforts (e.g. Bennett et al., 2011), and which this study has demonstrated continues to be a need (Moscovice & Rosenblatt, 2000; Wingert, Christianson, & Moscovice, 1991). Several challenges persist when surveying rural populations, such as the possibility of small sample sizes, limited data availability, the outright ability to find health service areas, as well as pre/post assessment difficulties. In addition, vaccine acceptability tends to coincide with whether or not an individual deems vaccination to be relevant at a certain point in time (Behrmann, 2011; Kata, 2012). Furthermore, with the rise of using the internet as a source for health information, misinformation and queries used (such as ‘vaccination’ versus ‘immunization,’ see Wolfe & Sharp, 2005), further challenges exist in developing interventions promotion efforts.

To address potential challenges faced by rural college students, H1 addressed the potential family, social, school, and work demands and their association with attitudes, subjective norms, and PBC related to vaccination intent. Attitudes, intent, and PBC towards getting vaccinations were related to work and school demands. This finding is notable, given that education research has noted that, for rural students working towards their education while also facing the challenges of working and paying for that education (e.g. Scott et al., 2016), healthcare needs can prove to be difficult, particularly in locations where provider access is lower (Rosenbaum et al., 2009). As such, it stands to reason that finding the time and access to healthcare facilities can be a challenge. This is coupled with the challenges that vaccines like HPV place might present for rural students, which, while widely recommended for the demographic, requires multiple visits to complete. H1 was partially supported, but findings represent multiple areas for researchers to address, both in scholarly and pragmatic work.

H2 suggested that attitudes, norms, and PBC would affect the vaccination intent. Attitudes and subjective norms were stronger predictors of vaccination intent, while PBC was not. However, a number of disparities in health services (e.g. one major provider in the town) exist in the area, so a replication of the study may yield different results. As such, while PBC did not contribute towards the model, past models have also only found little support, indicating that when added with other variables, its contribution is limited. This partial support for H2 suggests that developing interventions or educational materials that target attitudes and relationships (e.g. friends, family) towards rural students could be beneficial in improving vaccination rates, since they target individual values, as opposed to instituting mandates. Recent surveys have found that young adults tend to be less supportive of mandatory vaccine practices, including immunizations that are given at childhood (National Geographic, 2015; Pew Research Center, 2009, 2015), despite the fact that research indicates that mandates help to ensure vaccination (Looper et al., 2017). Partnerships at rural colleges could be helpful if they promote vaccines that might be offered at reduced costs or those that are free on campus.

Finally, RQ1 inquired about the vaccines rural college students receive. The most notable finding is that, though it is widely recommended, the HPV vaccine was only received or started by 14.2% of men and 30.2% of women. This is notable, given that it has largely been marketed towards young adults, as they are one of the prime demographics for the vaccine (Polonijo & Carpiano, 2013; Wong, 2008). Barriers to the vaccine, such as cost (Paiva et al., 2013) might further inhibit rural students’ ability to begin and complete the vaccine. Moreover, the issue of healthcare access might be evident here, given that access is an issue in rural and frontier communities, so researchers, educators, and healthcare practitioners ought to address knowledge, awareness and education of the importance of vaccines (e.g. Bednarczyk et al., 2017).

## Limitations and future directions

Potential limitations in the study include the use of a survey method via self-report. In the current study, this was a deliberate choice, particularly since the population surveyed was located in a remote location in the country with the closest major city being approximately 300 miles from the college. Healthcare providers are scarce and while advances in technology have made it possible to grant access to hard-to-reach populations, much work is needed to improve vaccination uptake among young adults. In a rural community, where the sample size may be smaller and it may be more difficult to get the necessary access to data, we recommend that data like this be used as an informative point to assist with the next steps in a larger line of work, such as an intervention to provide educational materials to improve vaccination screenings, or educational campaigns partnering with local clinics.

Notably, the study had a higher proportion of Native American students (8.0%) compared to non-rural institutions. In some cases, rural Native Americans have been involved in partnerships to improve vaccines, so the data on the larger population of respondents provides value for future research lines. However, beyond this study alone, in the United States, it would be worth developing partnerships with tribes and communities to build culturally appropriate methods to assess vaccination uptake and wellness, while also preserving rich traditions. It is likewise notable that because the study deliberately focused on rural college students and participation included those from ages 18 and above, future studies should take other approaches to focus on different populations in rural areas. These might include rural populations from ages 18–24, those under the age of 18, and those in varying socioeconomic statuses. Such insight could yield valuable information on vaccination intent and behavior and contribute towards developing improved healthcare programs and promotional efforts.

Examining the underlying attitudes, normative beliefs and perceived control, and subsequent behaviors of young adults—for whom these factors will largely persist throughout their lives—can provide a profound impact in delivering positive health intervention efforts. In addition, examining the beliefs of young adults in rurally located areas may reveal valuable information about the potential for communication strategies to be applied in future interventions that can help to increase behaviors. This is particularly notable, since current research has continued to show that minorities are less likely to receive vaccines (e.g. HPV) than White populations (Cohen & Legg, 2014). Through new health curricula, intervention efforts, grassroots campaigns and interpersonal communication efforts can improve vaccine behavior (National Vaccine Program Office, 2015). For instance, awareness of vaccines has been varied among adult populations, with self-reported vaccination coverage being suboptimal (Lu et al., 2017).

## Conclusion

Practically, this study contributes to a line of research in several ways. First, for rurally located young adults, attitudes and normative beliefs are predictors of vaccination intent, which is similar to research in other contexts, yet highlights a necessity to build education and resources for more remote communities. Second, control factors (PBC), which may be a result of a lack of resources, can be addressed in additional research and programs to overcome barriers to vaccinations. Developing health interventions that move towards serving medically underserved communities will be crucial in improving overall medical programs across the country. Researchers and practitioners can work to create better programs through surveying appropriate populations, developing interventions and partnering with appropriate organizations to improve or build programs that can improve vaccination uptake in poorer communities. Moreover, research efforts must take mixed-methods approaches in studying various demographics and their beliefs towards vaccines. Subsequently, scholarship should then develop health interventions at the grassroots-level to better address the needs of communities. In doing so, practitioners can better maximize opportunities for the potential of preventative health today.
